# Bronchopleural fistula and bilateral pneumothorax in a patient with COVID‐19

**DOI:** 10.1002/ccr3.5149

**Published:** 2021-11-25

**Authors:** Sangam Shah, Prince Mandal, Rajan Chamlagain, Rukesh Yadav, Yubraj Pande, Sanjit Kumar Sah, Basanta Sharma Paudel, Madan Gyawali

**Affiliations:** ^1^ Maharajgunj Medical Campus Institute of Medicine Tribhuvan University Maharajgunj Nepal; ^2^ Tribhuvan University Teaching Hospital Maharajgunj Nepal; ^3^ Department of Internal Medicine Institute of Medicine Tribhuvan University Maharajgunj Nepal

**Keywords:** bronchopleural fistula, COVID, hydropneumothorax, pneumothorax, SARS‐CoV‐19

## Abstract

COVID‐19 pneumonia causes several complications that include pneumothorax, hydropneumothorax, empyema, and rarely leads to bronchopleural fistula (BPF). BPF is a communication between the pleural space and the bronchial tree. We report a case of 24 years man with pneumothorax, hydropneumothorax, and BPF that appeared after COVID‐19 infection.

## Graphical abstract

Bronchopleural fistula may develop in a patient with COVID‐19 along with pneumothorax and hydropneumothorax.

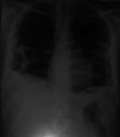

## 1 INTRODUCTION

Since its emergence, coronavirus disease (COVID‐19) has been a global pandemic. It is caused by the severe acute respiratory syndrome coronavirus 2 (SARS‐CoV‐2) that is known to cause severe respiratory dysfunction including pneumonia, acute respiratory distress syndrome (ARDS), and sepsis. The complications of COVID‐19 pneumonia include pneumothorax, hydropneumothorax, empyema, and rarely lead to bronchopleural fistula (BPF). BPF is a communication between the pleural space and the bronchial tree (main stem, lobar, or segmental bronchus). BPF commonly occurs after pulmonary resection (pneumonectomy, lobectomy, and segmentectomy) and is less common after infection. It has been estimated that BPF has a significant morbidity rate ranging from 25% to 71% following pneumonectomy or other pulmonary resection.^1^ Here we discuss a case of 24 years male with pneumothorax, hydropneumothorax, and BPF that appeared after COVID‐19 infection.

## 2 CASE PRESENTATION

A 24‐year‐old man was brought to our center with chief complaints of shortness of breath and cough for 6 days. He had shortness of breath even on walking on the level ground. The cough was copious and productive while sputum was yellowish. He had chest pain on right side that aggravated with coughing. He had no history of palpitation, loss of consciousness, lower limb swelling, and fever. He had COVID‐19 one month back that was resolved after 25 days of treatment. He had no known comorbidities. He neither smoked nor consumed alcohol.

On examination, patient was ill looking, conscious, and was well oriented to time, place, and person. He had pulse rate 110 beats/minute, respiratory rate 25/min, blood pressure 124/80 mm of Hg, and temperature was 98°F. He had no pallor, icterus, lymphadenopathy, cyanosis, or clubbing. There was bilateral decrease in air entry on chest auscultation over 4th and 5th intercostal spaces which was more on the left side. He had bronchial breath sounds bilaterally.

His laboratory investigations revealed hemoglobin 11.8 gm %, total leucocyte count 23,000/mm^3^, neutrophils 82%, lymphocytes 11%, and platelet count 508,000/mm^3^. Prothrombin time (PT) and international normalized ratio (INR) were 16 s and 1.22, respectively. His random blood sugar level was 3.7 mmol/L. The level of urea (10.7 mmol/L) and creatinine (215 µmol/L) was raised in the blood. He had elevated level of AST (86 U/L), ALT (221 U/L), and LDH (960 U/L) but decreased level of albumin (23 gm/L) and total protein (44 gm/L) in the blood.

**FIGURE 1:**
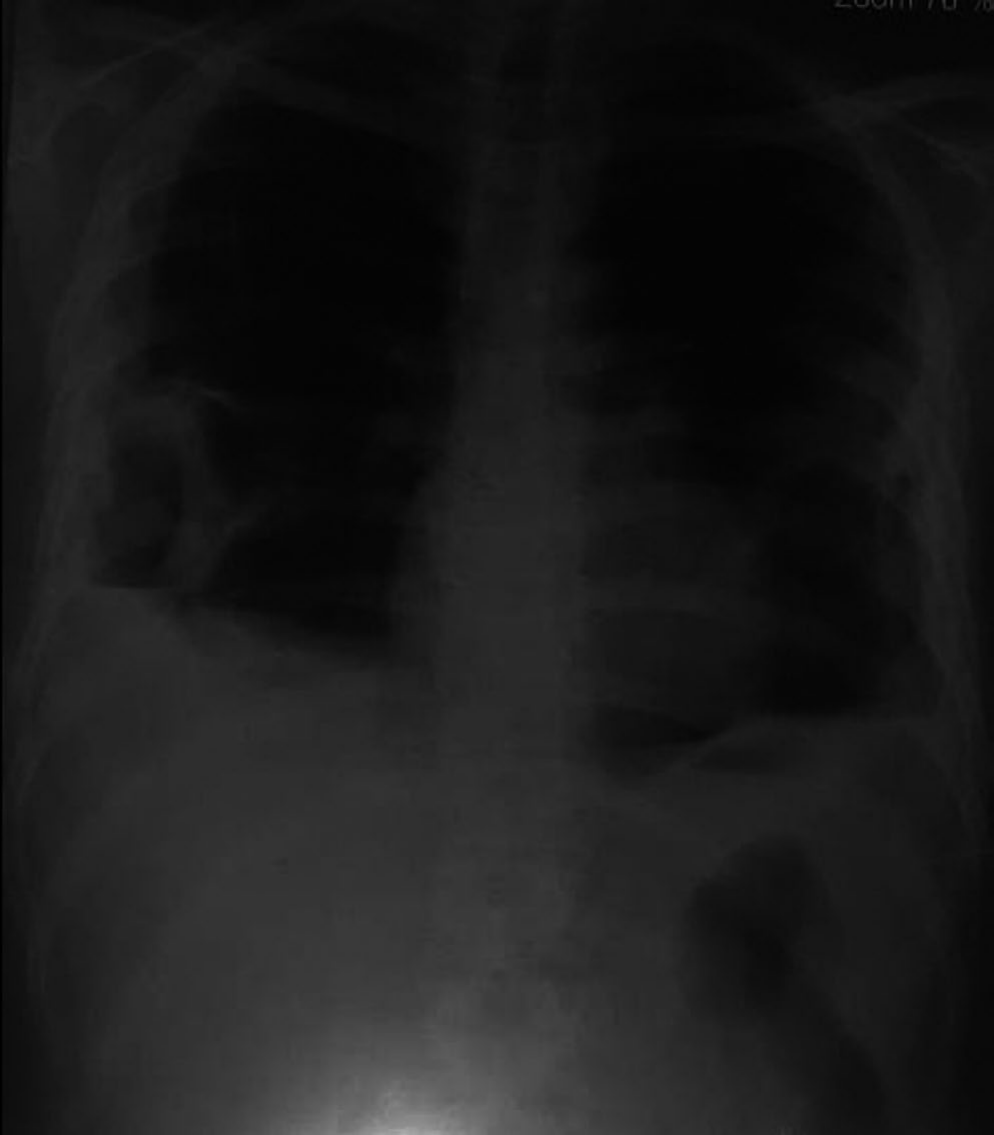
Chest X‐ray showing bilateral consolidation of lungs

Chest X‐ray showed bilateral consolidation of lungs in the middle zone (Figure 1). High‐resolution computed tomography (HRCT) of chest revealed bilateral multiple cavitary lesions with diffuse patchy ground‐glass opacification, thickened interlobular septa, bilateral lower lobe consolidation, traction bronchiectatic changes, and bilateral pleural effusion with passive atelectasis and mediastinal lymphadenopathy. There was cystic bulla communicating with pleura in the left side. Pleural fluid tapping was done under aseptic conditions with ultrasonography (USG) guidance, and 18 ml of serosanguinous fluid was aspirated. However, there was no growth of bacteria in it.

Based on the radiological findings, diagnosis of left‐sided hydropneumothorax, right‐sided pneumothorax, and bronchopleural fistula (BPF) on left side was made. Following this, bilateral chest tube was inserted to treat left side hydropneumothorax and right side pneumothorax. He developed fever after 5 days of hospital stay for which culture of the pleural fluid was done that revealed the growth of *Acinetobacter lwoffi*. He was treated with (piperacillin +tazobactum [4.5 g IV QID], linezolid [600 mg BD], montelukast [10 mg OD], and hydrocortisone [100 mg IV BD]) for 14 days. Repeat chest X‐ray was normal following which, chest tube was removed. He improved clinically and was discharged on oral medications (amoxicillin and clavulanic acid [625 mg TDS] for 7 days, azithromycin [500 mg OD] for 5 days, and montelukast [10 mg OD] for 7 days). His follow‐up was not eventful.

## 3 DISCUSSION

Necrotizing lung infections, post‐lung resection, and chemoradiation therapy are the common causes of BPF.^1^ The overall mortality rate of BPF is between 25% and 71%.^1^ Pleural space infection, hypoxia, and inadequate lung expansion are common complications of recurrent air leakage.^1^ The pathophysiology of BPFs in SARS‐CoV‐2 is unknown; however, there are few case reports describing cavitary lung lesions as a cause of BPFs.^2^ Intra‐alveolar bleeding, which leads to additional parenchymal cell necrosis, as corroborated by postmortem studies can also be correlated with the cavitation in the lungs.^3^

The radiological investigation of choice for diagnosing BPF is contrast‐enhanced chest computed tomography.^4^ Bronchoscopy is the gold standard for diagnosing and localizing BPF, with progressive balloon occlusion allowing for real‐time assessment of air leak site.^4^, ^5^ Because persistent BPF is usually caused by pleural infection, the choices of treatment are chest tube drainage and long‐term antibiotics.^4^ Large BPFs (>8 mm in diameter) are treated surgically.^4^ Endobronchial intervention is preferred in high‐risk surgical candidates to avoid the risk of surgery and for management of smaller fistulas.^5^ Patients with BPF due to causes other than lung resection, such as our patient with SARS‐CoV‐2 pneumonia, are treated with bronchoscopy with endobronchial valves, as surgical closure of a BPF is likely to fail due to friable lung tissue.^4^, ^5^ Endobronchial procedures may also include the use of sealing agents such as glues or an autologous blood patch.

Endobronchial occlusion is a less invasive alternative to thoracic surgery for treating BPFs.^6^, ^7^ However, the treatment of pneumothorax or empyema along with bronchial fistula secondary to COVID‐19 pneumonia has not been discussed in literatures. In our situation, the chest tube effectively drained pleural cavity without a septal wall. As a result, pleural cavity debridement surgery was not done.

## 4 CONCLUSION

Bronchopulmonary fistula is one of the rare complications of COVID‐19. Clinicians should be aware of this scenario, which can be treated with bronchoscopy procedures like EBV or sealing the fistula with glues or blood patch or even surgery for severe cases. In our patient, chest tube was sufficient for the treatment without the complications.

## CONFLICT OF INTEREST

None.

## AUTHOR CONTRIBUTIONS

SS conceptualized the study, reviewed, edited the manuscript, and charged the case. PM and SS wrote the original, reviewed and edited the manuscript. SS, PM, RC, RY, YP, SKS, BSP, and MG charged the case and reviewed the manuscript.

## CONSENT

Written informed consent was obtained from the patient.

## Data Availability

All the required information is in manuscript itself.
